# Agglomeration of Luminescent Porous Silicon Nanoparticles in Colloidal Solutions

**DOI:** 10.1186/s11671-016-1593-4

**Published:** 2016-08-19

**Authors:** Kateřina Herynková, Miroslav Šlechta, Petra Šimáková, Anna Fučíková, Ondřej Cibulka

**Affiliations:** 1 Department of Thin Films and Nanostructures, Institute of Physics, Academy of Sciences of the Czech Republic, v.v.i, Cukrovarnicka 10, CZ-162 53 Prague, Czech Republic; 2 Division of Biomolecular Physics, Institute of Physics of Charles University, Prague, Czech Republic; 3 Department of Chemical Physics and Optics, Faculty of Mathematics and Physics, Charles University, Prague, Czech Republic

**Keywords:** Nanocrystalline silicon, Porous silicon, Nanoparticles, Colloids, Agglomeration, 78.67.Rb, 78.67.-n, 87.85.Qr, 87.85.Rs, 81.07.-b

## Abstract

We have prepared colloidal solutions of clusters composed from porous silicon nanoparticles in methanol, water and phosphate-buffered saline (PBS). Even if the size of the nanoclusters is between 60 and 500 nm, due to their highly porous “cauliflower”-like structure, the porous silicon nanoparticles are composed of interconnected nanocrystals having around 2.5 nm in size and showing strong visible luminescence in the orange-red spectral region (centred at 600–700 nm). Hydrophilic behaviour and good solubility of the nanoclusters in water and water-based solutions were obtained by adding hydrogen peroxide into the etching solution during preparation and 16 min long after-bath in hydrogen peroxide. By simple filtration of the solutions with syringe filters, we have extracted smaller nanoclusters with sizes of approx. 60–70 nm; however, these nanoclusters in water and PBS solution (pH neutral) are prone to agglomeration, as was confirmed by zeta potential measurements. When the samples were left at ambient conditions for several weeks, the typical nanocluster size increased to approx. 330–400 nm and then remained stable. However, both freshly filtered and aged samples (with agglomerated porous silicon nanoparticles) of porous silicon in water and PBS solutions can be further used for biological studies or as luminescent markers in living cells.

## Background

Nanocrystalline silicon has been studied for the 20 years not only due its potential use in silicon nanophotonics or for enhancing solar energy conversion [1] but also in biological and medical applications. Silicon nanoparticles extracted from highly porous silicon (Si-ncs) showing visible room-temperature luminescence have been suggested to be used, e.g. as fluorescent labels, biological sensors, photoresponsive systems for regulated drug delivery or scaffold for various tissues [2–5]. Photo- and sono-senzitizing properties of porous silicon were successfully employed in simultaneous cancer therapy and diagnostics (http://dx.doi.org/10.1007/s00340-011-4562-8) [6, 7], and efficient uptake of the nanoparticles by cancer cells was demonstrated in vitro [8]. Si-ncs are explored as in vivo imaging agents [9, 10], too. The main advantages of Si-ncs are low cytotoxicity [11], easy functionalization [12], efficient photoluminescence [13] and bio-degradability (http://dx.doi.org/10.1016/j.nano.2016.04.004) [14, 15]. However, many requirements should be fulfilled in order to produce nanocrystals suitable for biological research such as relevant size and narrow size distribution, good dispersability in the cell environment and good long-term stability.

For biological in vivo studies, perhaps even for the use of silicon nanoparticles for controlled drug delivery, suitable samples are colloidal solutions in water-based (non-toxic) or isotonic solvents such as phosphate-buffered saline (PBS); alcohol solutions in methanol or ethanol must be strongly diluted because of their toxicity for living cells. Further requirement is that the nanoparticles must have suitable size of the order of units, tens or maximally couple of hundreds nanometers [16] in order to easily penetrate into the cells via endocytosis process. Last but not least, the Si-ncs have to be hydrophilic (easily soluble in water) and stable in time, without any agglomeration tendency.

In this paper, we report on colloidal solutions of Si-ncs in methanol, water and PBS. Methanol was used for comparison and easy solubility of the Si-ncs in it; however, an intentional solvent was PBS because it is a non-toxic and isotonic buffer solution commonly used in biological research and medicine. Native hydrophobicity of porous silicon was overcome by modification of the preparation conditions—a small amount of hydrogen peroxide added to the etching bath, and short after-bath in hydrogen peroxide causes a switch to hydrophilic behaviour of the Si-ncs. We address here the problem of the agglomeration of the nanocrystals in the colloidal solutions. Hydrophilic oxidized Si-ncs tend to agglomerate due to their physical properties, such as zeta potential, but it is relatively a long process, it takes its course in the time interval of several weeks.

## Methods

**Fig. 1:**
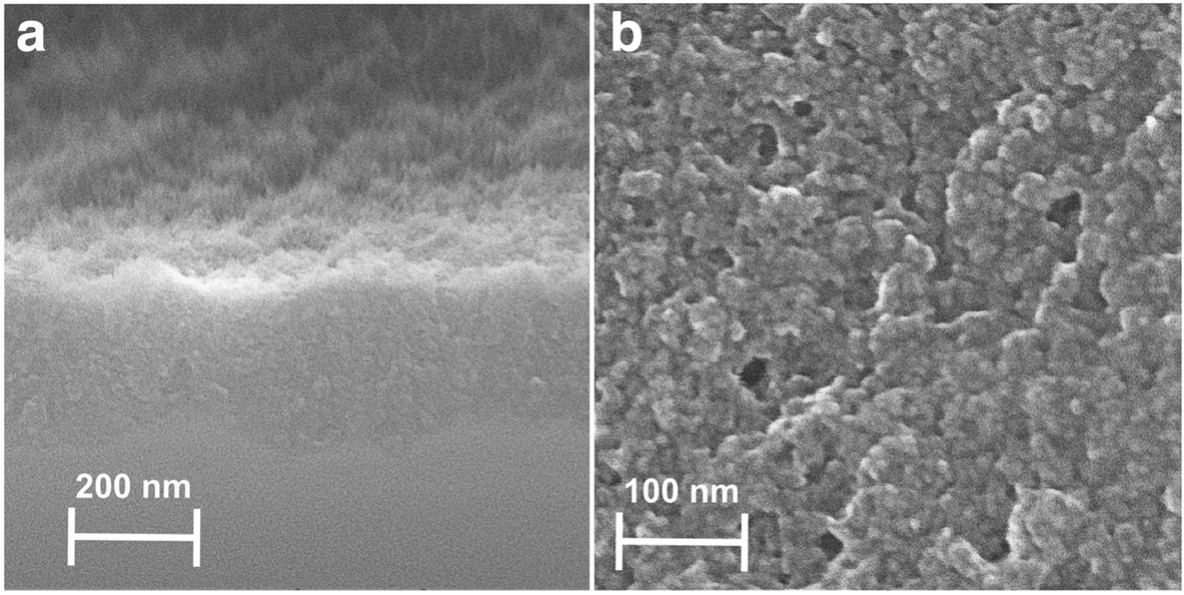
SEM images of a “standard” porous silicon layer before mechanical scraping from the silicon wafer: **a** Cross-section of the wafer showing a ~250-nm thick porous silicon layer. **b** Typical image of the porous silicon surface

Luminescent porous silicon nanoparticles were prepared by standard anodic electrochemical etching of a p-type [100] silicon wafer (boron doped, resistivity of 0.06–0.1 Ωcm) in a 1:3 aqueous-HF (49 %) solution in ethanol for 2 h at a current density 1.6 mA/cm^2^. The etching produces a ~250-nm thick layer of highly porous silicon (see Fig. 1a) which has a typical cauliflower structure clearly visible in scanning electron microscopy (SEM) image in Fig. 1b. However, such “standard” porous silicon prepared by the above-mentioned and typically used preparation conditions is hydrophobic, and its solubility in water and water-based isotonic solutions including PBS is very limited. Hydrophilic behaviour of the porous silicon can be achieved by slightly modifying the etching conditions: During etching of the second type of porous silicon (which will be further denoted as “white” due to white-ivory colour of the samples), higher current density (2.5 mA/cm^2^) was used, a small amount of 30 % hydrogen peroxide was added to the electrolyte (2 ml of H_2_O_2_ in total 50 ml of electrolyte) and the resulting porous silicon sample was then post-etched in a hydrogen peroxide bath for 16 min. These preparation conditions are empirically optimised and lead to the highest effect on the infrared and photoluminescence spectra compared to the samples obtained by the “standard” etching procedure.

Porous silicon powders were then mechanically scraped from the etched wafers, and after ultrasonic pulverization (band ultrasonic finger of 2.5 mm in diameter) and optional filtering by 1-μm syringe filters, the dispersions in methanol, water or PBS were obtained. Starting nanoparticles’ concentration was always 2 mg of Si-ncs powders in 1 cm^3^ of solvent. More detailed description of the preparation procedure and characterization of both types of samples by transmission electron microscopy and X-ray diffraction can be found in our previous publications [16–18].

Scanning electron microscopy (SEM) was performed using an electron microscope Tescan Maia3 with the electron bias of 10 keV. The size of Si nanoparticles in colloidal suspensions was determined by dynamic light scattering (DLS) measurement using a Zetasizer Nano ZS (Malvern), the samples being illuminated by the 633 nm line of a He-Ne laser and signal was detected in a backscattering geometry. The dynamic nanocluster sizes were calculated by Zetasizer software in General purpose (normal) resolution mode. The apparatus was equipped by a zeta potential and titrator unit (MPT-2). The titration unit enables to change the pH of the sample by adding small amounts of 0.2 or 0.02 M HCl and 0.25 M NaOH to the sample, and zeta potential is measured at individual pH points. The pH dependence of zeta potential was measured both in the acid-to-base and base-to-acid direction.

Fourier transform infrared (FTIR) spectra were recorded by a Thermo Scientific Nicolet iN10 infrared microscope. Photoluminescence (PL) was excited by a 325-nm line of a continuous wave HeCd laser and detected by imaging spectrograph connected to an Andor CCD camera. All spectra were recorded at room temperature and corrected for the spectral response of the detection path.

## Results and Discussion

**Fig. 2:**
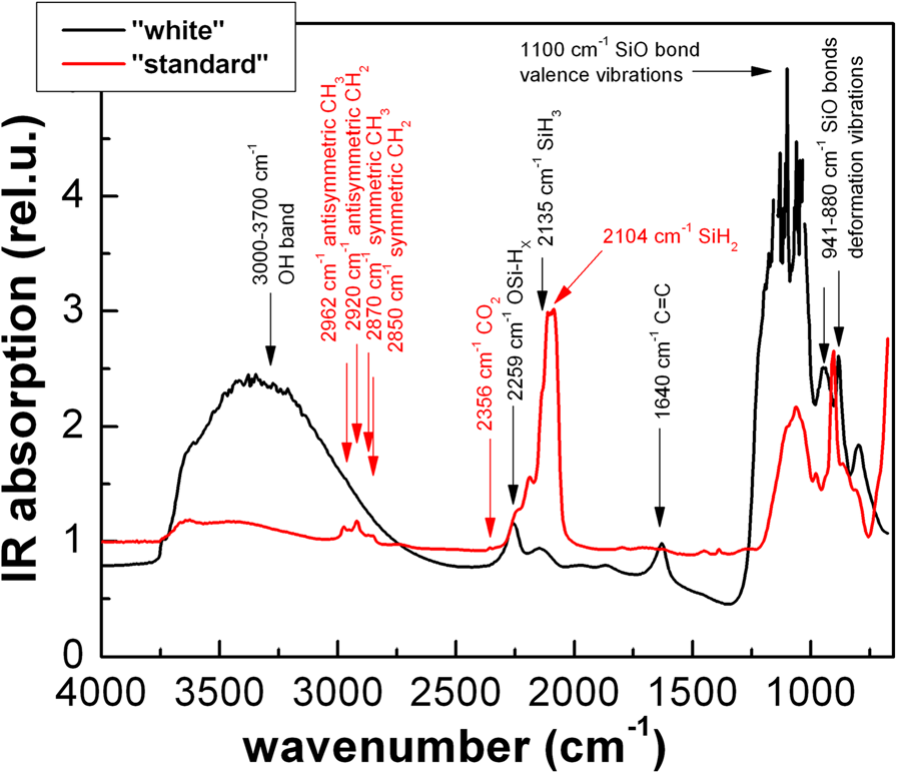
Comparison of FTIR spectra of “standard” and “white” porous silicon. The vibrations attributed to the particular spectral lines are indicated in the graph

Figure 2 showing FTIR spectra of both types of samples—native “standard” and “white” porous silicon layers attached to the silicon wafers, before mechanical scraping from the substrate—demonstrates the basic difference between these samples. While “standard” porous silicon sample exhibits a clear peak at 2104 cm^−1^ attributed to the presence of Si-H_2_ bonds and a peak at 2135 cm^−1^ attributed to the presence of Si-H_3_ bonds, a series of peaks at around 880–1100 cm^−1^ due to valence and deformation vibrations of Si-O bonds is more pronounced in “white” porous silicon. Carbon-related vibrations around 2800–3000 cm^−1^, 2356 cm^−1^ and 1640 cm^−1^ as well as OH band above 3000 cm^−1^ are likely related to the remnants of the ethanol etching solution and are not of interest. Therefore, silicon nanocrystals composing “white” porous silicon have more strongly oxidized surface than partially hydrogenized and partially oxidized “standard” ones (OSi-H_x_ vibration at 2259 cm^−1^ is present in both types of samples).

**Fig. 3:**
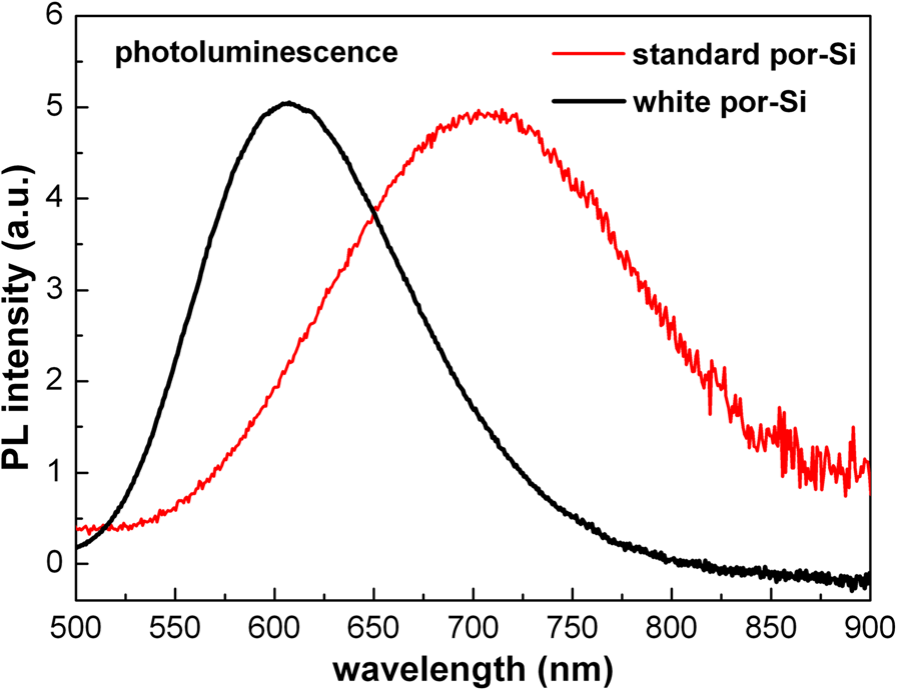
Typical photoluminescence spectra of “standard” and “white” porous silicon

The nanocrystal core of “white” porous silicon is smaller which is implied by the blue shift of photoluminescence (Fig. 3) due to the stronger quantum confinement effect—the PL of “standard” porous silicon is red (peaked at 700 nm), while the PL of “white” porous silicon is light orange and centred at around 600 nm. More complex PL studies and discussion of the PL origin can be found in Refs. [19–23].

**Fig. 4:**
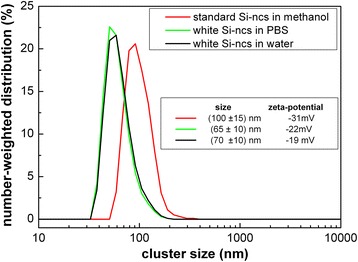
Hydrodynamic diameters of selected freshly prepared and filtered silicon nanocrystals in colloidal solutions determined by dynamic light scattering, and their zeta potentials

Figure 4 compares the hydrodynamic diameters of selected freshly prepared and filtered colloidal solutions determined by DLS together with corresponding zeta potentials. In alcohol solutions (an example is given by the red line in Fig. 4 corresponding to “standard” Si-ncs in methanol), the filtration by syringe filters of both kinds of samples is easy and it is possible to obtain nanoclusters with typical size of about 100 nm. Zeta potential of all the samples and solvents was negative, i.e. Si-ncs in solutions are surrounded with negative charge. However, the negative charge must be sufficiently high in order to repel the nanoclusters from each other; the limit where electrostatic potential on the nanocrystals’ surface becomes sufficient to repel the nanoclusters from each other and prevent agglomeration does not depend on the particles’ material and is around −30 mV. Both kinds of Si-ncs in the methanol solutions are reasonably stable; their zeta potential gets slightly below −30 mV (results for “white” porous silicon in methanol are not shown, but are similar to the methanol solutions of “standard” Si-ncs). Water-based colloidal solutions, however, are possible only to make with hydrophilic “white” Si-ncs. The obtained Si-ncs’ size is smaller, approx. 60–70 nm; however, the filtration yield is substantially lower than in alcohol solutions. Moreover, zeta potential around −20 mV indicates a tendency of the nanoclusters to agglomerate in the solution.

**Fig. 5:**
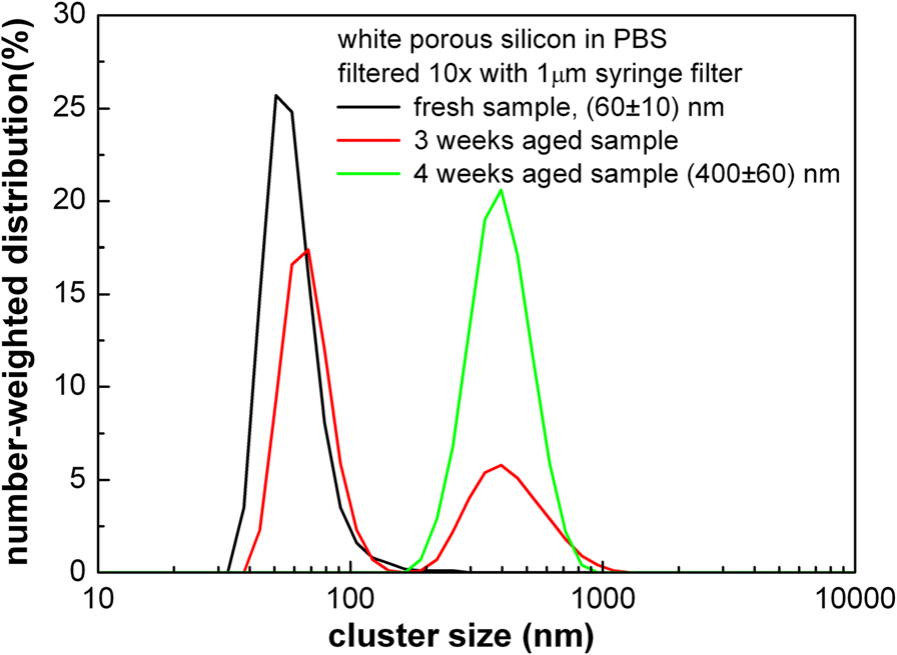
Ageing of the “white” porous silicon in PBS solution. DLS measurement of the freshly filtered sample and the same sample left for 3 and 4 weeks at ambient conditions

The agglomeration of “white” Si-ncs in water-based colloidal solutions—the increase of the nanoclusters’ size in time—becomes apparent from Fig. 5: The freshly prepared and filtered sample of “white” porous silicon in PBS solution is composed of ~60-nm nanoclusters. If the sample is left for several weeks at ambient conditions, the nanoclusters will get bigger and form 300–400-nm sized aggregates. In order to avoid this agglomeration in subsequent biological research, either only freshly prepared colloidal solutions can be used or there are a few possibilities to stabilise the solutions: First, it is possible to vary slightly the pH of colloidal solutions in order to increase the absolute value of the zeta potential (providing that the solution is still not toxic for living cells due to pH); the second possibility is the stabilization of nanocrystals via passivating their surface with simple organic compounds.

**Fig. 6:**
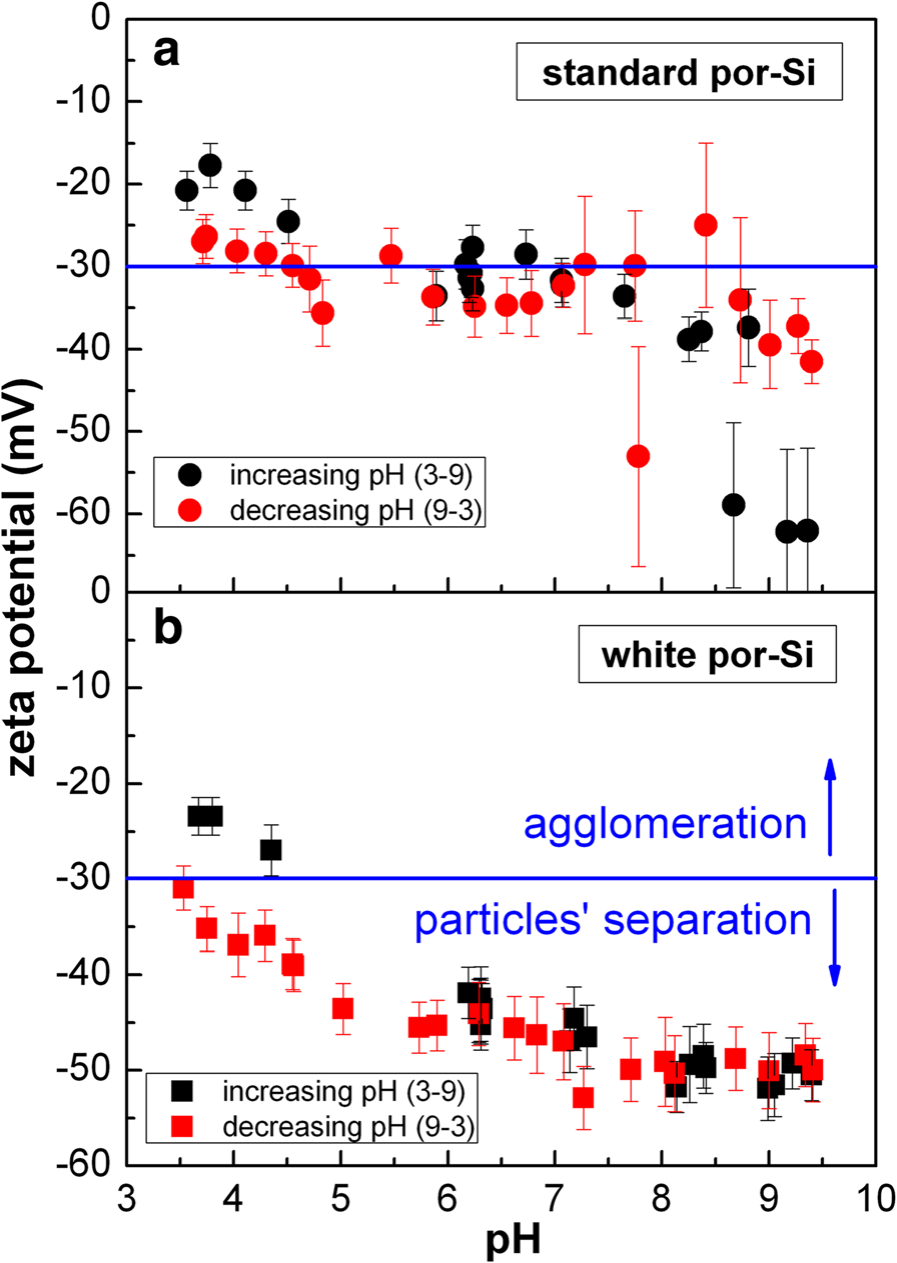
pH dependence of zeta potential of unfiltered (**a**) “standard” and (**b**) “white” porous silicon colloidal solutions in water. The *blue line* indicates a −30 mV limit below which the particles in solutions are stable (repel electrically from each other)

Figure 6 shows the pH dependencies of zeta potential of both “standard” and “white” Si-ncs in colloidal solutions (non-filtered samples, initially in water). Both curves decrease with increasing pH which is a well-known fact—acids with excess of positive ions decrease the negative charge on the surface of the nanoclusters while bases with excess of negative OH^−^ groups increase the Si-ncs’ negative charge. For the sake of biological and medicinal studies, the region of interest is neutral pH between 5.5 and 7.5, where is plateau. In this central region, the zeta potential of “standard” Si-ncs gets around the limit value of −30 mV. On the other side, the whole curve for “white” Si-ncs is shifted to lower zeta potentials, i.e. to the region with good solution stability. Corresponding hydrodynamic size of the nanoclusters is about 330 nm for both types of porous silicon.

Therefore, “white” Si-ncs are not only much more suitable for the preparation of isotonic colloidal solutions due to their hydrophilicity but also the solutions of Si-ncs with sizes of ~330 nm reveal much better stability in time. “White” Si-ncs whose surface si to the higher degree oxidized – negatively charged oxygen atoms on the Si-ncs’ surface make more negative electric charge than positively charged hydrogen atoms in “standard” porous silicon samples. A series of the samples with graded amount of oxygen is now being produced, and further study of their zeta potentials is in progress.

## Conclusions

Colloidal dispersions of porous silicon nanocrystals in methanol, water and PBS show visible luminescence peaked at 600–700 nm in dependence on the etching conditions. “White” Si-ncs are hydrophilic and more suitable for preparing colloidal solutions for biological research. In freshly prepared, ultrasonicated and filtered solutions, it is possible to obtain Si-nc clusters of ~60 nm in size; however, they agglomerate, and in the time horizon of several weeks, their size increases to 300–400 nm. Colloidal solutions of “white” Si-ncs of that increased size remain stable and can be subsequently used for biological studies (cytotoxicity, fluorescent labels for single molecule detection in the cell).

## Abbreviations

DLS: Dynamic light scattering
FTIR: Fourier transform infrared (spectra)
HCl: Hydrochloric acid
HF: Hydrofluoric acid
NaOH: Sodium hydroxide
PBS: Phosphate-buffered saline
PL: Photoluminescence
SEM: Scanning electron microscopy
Si-ncs: Silicon nanocrystals

## References

[1] Sailor MJ (2011). Porous silicon in practice: preparation, characterization and applications.

[2] Santos HA (2014). Porous silicon for biomedical applications.

[3] Fučíková A, Valenta J, Pelant I, Kůsová K (2011). Nanocrystalline silicon in biological studies. Phys Status Solidi C.

[4] Amrita P, Avijit J, Karthik S, Manoranjan B, Zhao Y, Pradeep Singh ND (2016). Photoresponsive real time monitoring silicon quantum dots for regulated delivery of anticancer drugs. J Mater Chem B.

[5] Erogbogbo F, Yong KT, Roy I, Xu GX, Prasad PN, Swihart MT (2008). Biocompatible luminescent silicon quantum dots for imaging of cancer cells. ACS Nano.

[6] Xiao L, Gu L, Howell SB, Sailor MJ (2011). Porous silicon nanoparticle photosensitizers for singlet oxygen and their phototoxicity against cancer cells. ACS Nano.

[7] Timoshenko VYu: Porous silicon for cancer theranostics applications [abstract], 9^th^ International Conference Porous Semiconductors – Science and Technology, Alicante-Benidorm, Spain, March 2014, p. 182

[8] Osminkina LA (2015). Porous silicon nanoparticles as efficient sensitizers for sonodynamic therapy of cancer. Microporous Mesoporous Mater.

[9] Hessel CM, Rasch MR, Hueso JL, Goodfellow BW, Akhavan VA, Puvanakrishnan P, Tunnel JW, Korgel BA (2010). Alkyl passivation and amphiphilic polymer coating of silicon nanocrystals for diagnostic imaging. Small.

[10] Tu C, Ma X, House A, Kauzlarich SM, Louie AY (2011). PET imaging and biodistribution of silicon quantum dots in mice. ACS Med Chem Lett.

[11] Fučíková A, Valenta J, Pelant I, Hubálek Kalbáčová M, Brož A, Rezek B, Kromka A, Bakaeva Z (2014). Silicon nanocrystals and nanodiamonds in live cells: photoluminescence characteristics, cytotoxicity and interaction with cell cytoskeleton. RSC Adv.

[12] Secret E, Smith EK, Dubljevic V, Moore E, Macardle P, Delalat B, Rogers ML, Johns TG, Durand JO, Cunin F, Voelcker NH (2013). Antibody-functionalized porous silicon nanoparticles for vectorization of hydrophobic drugs. Adv Healthcare Mater.

[13] Canham LT (1990). Silicon quantum wire fabrication by electrochemical and chemical dissolution of wafers. Appl Phys Lett.

[14] Park JH, Gu L, von Maltzahn G, Ruoslahti E, Bhatia SN, Sailor MJ (2009). Biodegradable luminescent porous silicon nanoparticles for in vivo applications. Nat Mater.

[15] Low SP, Williams KS, Canham LT, Voelcker NH (2006). Evaluation of mammalian cell adhesion on surface-modified porous silicon. Biomaterials.

[16] Herynková K, Podkorytov E, Slechta M, Cibulka O, Leitner J, Pelant I (2014). Colloidal solutions of luminescent porous silicon clusters with different cluster sizes. Nanoscale Res Lett.

[17] Herynková, K, Podkorytov E, Šlechta M, Cibulka O: Stabilization of silicon nanoparticles in colloidal solutions. Phys. stat. sol. (c) 2016, to be published in April 2016

[18] Kůsová K, Cibulka O, Dohnalová K, Pelant I, Valenta J, Fučíková A, Lang J, Englich J, Matějka P, Štěpánek P, Bakardjieva S (2010). Brightly luminescent organically capped silicon nanocrystals fabricated at room temperature and atmospheric pressure. ACS Nano.

[19] Dohnalová K, Ondič L, Kůsová K, Pelant I, Rehspringer JL, Mafouana RR (2010). White-emitting oxidized silicon nanocrystals: discontinuity in spectral development with reducing size. J Appl Phys.

[20] Valenta J, Fučíková A, Pelant I, Kůsová K, Dohnalová K, Aleknavičius A, Cibulka O, Fojtík A, Kada D (2008). On the origin of the fast photoluminescence band in small silicon nanoparticles. New J Phys.

[21] Dohnalová K, Pelant I, Kůsová K, Gilliot P, Gallart M, Crégut O, Rehspringer JL, Honerlage B, Ostatnicky T, Bakardjeva S (2008). Closely packed luminescent silicon nanocrystals in a distributed-feedback laser cavity. New J Phys.

[22] Dohnalová K, Žídek K, Ondič L, Kůsová K, Cibulka O, Pelant I (2009). Optical gain at the F-band of oxidized silicon nanocrystals. J Phys D Appl Phys.

[23] Ondič L, Kůsová K, Ziegler M, Fekete L (2014). A complex study of the fast blue luminescence of oxidized silicon nanocrystals: the role of the core. Nanoscale.

